# Telehealth Experience Among Patients With Limited English Proficiency

**DOI:** 10.1001/jamanetworkopen.2024.10691

**Published:** 2024-05-09

**Authors:** Jorge A. Rodriguez, Elaine C. Khoong, Stuart R. Lipsitz, Courtney R. Lyles, David W. Bates, Lipika Samal

**Affiliations:** 1 Division of General Internal Medicine and Primary Care, Brigham and Women’s Hospital, Boston, Massachusetts; 2 Harvard Medical School, Boston, Massachusetts; 3 Division of General Internal Medicine, Department of Medicine, University of California San Francisco, San Francisco; 4 Center for Vulnerable Populations, Zuckerberg San Francisco General Hospital, University of California San Francisco, San Francisco; 5 Center for Healthcare Policy and Research, University of California Davis, Davis; 6 Department of Public Health Sciences, University of California Davis School of Medicine, Davis

## Abstract

This cross-sectional study assesses the implication of patients’ English language skills for telehealth use and visit experience.

## Introduction

Patients with limited English proficiency (LEP) face disparities in using telehealth.^1^ While research has focused on access, attention to patient experience is essential. Patients with LEP have worse experience with in-person care.^2^ We examined differences in telehealth access and experience between patients with LEP and patients with English proficiency (EP) in California.

## Methods

We analyzed the 2021 adult data from California Health Interview Survey (CHIS), which is conducted in 6 languages.^3^ The Brigham and Women's Hospital Institutional Review Board deemed this cross-sectional study exempt from review and waived informed consent because publicly available data were used. We followed the STROBE reporting guideline.

Study exposure was LEP, defined as speaking English not well or not at all. Study outcomes were telehealth use and visit experience. For telehealth use, CHIS participants were asked whether they had used video or telephone telehealth in the past 12 months (eAppendix in Supplement 1). For visit experience, participants were asked to compare their experience with video or telephone visits to in-person visits. We dichotomized visit experience to better or same vs worse. Outcomes of patients with LEP or EP were assessed and compared. Covariates included factors associated with use of digital tools: age, sex, marital status, insurance status, educational level, poverty level, health status, internet use, and having usual source of care.^1,4^ Self-reported race and ethnicity and metropolitan area residency were excluded due to collinearity.

We performed bivariable comparisons using weighted χ^2^ analysis. We then performed weighted multivariable logistic regression to ascertain odds of worse experience after controlling for covariates. We used survey-supplied replicate weights to produce population estimates, as recommended by CHIS.^3^ Weights represent California’s residential population.

Two-sided *P* < .05 was considered significant. Analyses were performed using R 3.6.2 (R Core Team).

## Results

The study included 24 453 participants (10 735 males [weighted 49%], 13 718 females [weighted 51%]), representing a population of 29 649 837. Patients with LEP accounted for 9% of participants and 7% of telehealth users. Telehealth users with LEP differed significantly from users with EP across most covariates (Table 1). Among telehealth users, patients with LEP accounted for 6.8% of video visit users (387, representing a population of 840 764) and 8.1% telephone visit users (484, representing a population of 1 021 909).

**Table 1.** Characteristics of Survey Participants With Video and Telephone Visits by English Proficiency

Characteristic and visit type	Participants, weighted No. (%)^a^		*P* value
	With EP	With LEP	
**Age, y**			
Video visits			
18-64	8 717 663 (76)	505 232 (60)	<.001
65-74	1 755 226 (15)	167 599 (20)	
75-84	787 812 (7)	114 950 (14)	
≥85	224 488 (2)	52 983 (6)	
Telephone visits			
18-64	8 507 065 (74)	645 847 (63)	<.001
65-74	1 827 401 (16)	184 689 (18)	
75-84	920 012 (8)	140 853 (14)	
≥85	270 200 (2)	50 520 (5)	
**Sex**			
Video visits			
Female	6 396 076 (56)	545 284 (65)	.003
Male	5 089 113 (44)	295 480 (35)	
Telephone visits			
Female	6 550 096 (57)	658 669 (64)	.009
Male	4 974 583 (43)	363 239 (36)	
**Marital status**			
Video visits			
Married	5 999 051 (52)	538 164 (64)	<.001
Telephone visits			
Married	5 956 321 (52)	678 494 (66)	<.001
**Educational level**			
Video visits			
<High school	860 900 (8)	568 207 (68)	<.001
≥High school graduate	10 624 289 (93)	272 558 (32)	
Telephone visits			
<High school	976 031 (9)	704 903 (69)	<.001
≥High school graduate	10 548 648 (92)	317 006 (31)	
**Poverty level, FPL, %**			
Video visits			
0-99	1 102 503 (10)	292 660 (35)	<.001
100-199	1 474 606 (13)	262 100 (31)	
200-299	1 356 158 (12)	126 409 (15)	
>300	7 551 922 (66)	159 596 (19)	
Telephone visits			
0-99	1 241 153 (11)	360 163 (35)	<.001
100-199	1 706 277 (15)	328 736 (32)	
200-299	1 468 320 (13)	168 390 (16)	
>300	7 108 928 (62)	164 620 (16)	
**Race and ethnicity^b^**			
Video visits			
Asian, non-Hispanic	1 333 440 (12)	224 768 (27)	<.001
Hispanic	3 350 989 (29)	587 792 (70)	
White, non-Hispanic	5 676 456 (49)	25 406 (3)	
Other^c^	1 124 303 (10)	2798 (0.3)	
Telephone visits			
Asian, non-Hispanic	1 221 175 (11)	274 510 (27)	<.001
Hispanic	3 624 612 (31)	716 985 (70)	
White, non-Hispanic	5 500 397 (48)	26 320 (3)	
Other	1 178 494 (10)	4094 (0.4)	
**Insurance status**			
Video visits			
Insured	11 229 096 (98)	789 494 (94)	<.001
Uninsured	256 093 (2)	51 270 (6)	
Telephone visits			
Insured	11 236 529 (97)	959 270 (94)	<.001
Uninsured	288 150 (3)	62 639 (6)	
**Usual source of care**			
Video visits			
With usual source	10 833 181 (94)	767 566 (91)	.02
Without usual source	652 008 (6)	73 198 (9)	
Telephone visits			
With usual source	11 236 529 (97)	959 270 (94)	<.001
Without usual source	288 150 (3)	62 639 (6)	
**Health status**			
Video visits			
Excellent	1 703 435 (15)	31 186 (4)	<.001
Very good	3 964 154 (35)	88 196 (10)	
Good	3 798 604 (33)	264 560 (31)	
Fair	1 627 917 (14)	363 597 (43)	
Poor	391 080 (3)	93 226 (11)	
Telephone visits			
Excellent	1 573 758 (14)	40 682 (4.0)	<.001
Very good	3 762 698 (33)	104 937 (10)	
Good	3 995 425 (35)	337 931 (33)	
Fair	1 759 393 (15)	426 887 (42)	
Poor	433 404 (4)	111 472 (11)	
**Internet use**			
Video visits			
Almost constantly	3 776 041 (33)	116 560 (14)	<.001
Many times a day	5 056 062 (44)	194 196 (23)	
A few times a day	1 865 051 (16)	246 469 (30)	
Less than a few times a day	798 928 (7)	272 646 (33)	
Telephone visits			
Almost constantly	3 546 005 (31)	140 291 (14)	<.001
Many times a day	4 956 503 (43)	272 695 (27)	
A few times a day	2 051 646 (18)	295 877 (29)	
Less than a few times a day	981 622 (9)	301 949 (30)	

Abbreviations: EP, English proficiency; FPL, federal poverty level; LEP, limited English proficiency.

^a^ Values represent population estimates for California’s residential population. Video sample size: EP: 11 485 1891; LEP: 840 764. Telephone sample size: EP: 11 524 6791; LEP: 1 021 9091.

^b^ Race and ethnicity were self-reported in the survey.

^c^ Other included non-Hispanic Black, American Indian or Alaskan Native, and other or 2 or more races.

In unadjusted analyses, patients with LEP were less likely to report either video or telephone telehealth use (37% vs 50%; *P* < .001). In adjusted analyses, patients with LEP were less likely to report video or telephone telehealth use (odds ratio [OR], 0.63; 95% CI, 0.52-0.77; *P* < .001) vs patients with EP (Table 2). For video visits, in unadjusted analyses, patients with LEP reported worse experience (32% vs 26%; *P* = .04) vs patients with EP. In adjusted analyses, patients with LEP were more likely to report worse experience with video visits than in-person visits (OR, 1.42; 95% CI, 1.04-1.94; *P* = .03). For telephone visits, there was no difference in visit experience between the 2 groups (unadjusted: 29% vs 31%, *P* = .60; adjusted: OR, 1.24 [95% CI, 0.91-1.69], *P* = .17).

**Table 2.** Telehealth Use and Visit Experience vs In-Person Visits by English Proficiency

	Unadjusted OR (95% CI)^a^	*P* value	Adjusted OR (95% CI)^a^^,^^b^	*P* value
Telehealth use	0.60 (0.52-0.70)	<.001	0.63 (0.52-0.77)	<.001
Experience with telehealth visits vs in-person visits				
Worse experience with telephone visits	1.10 (0.82-1.50)	.56	1.24 (0.91-1.69)	.17
Worse experience with video visits	1.35 (1.01-1.81)	.04	1.42 (1.04-1.94)	.03

Abbreviation: OR, odds ratio.

^a^ English-proficient patients served as the reference group.

^b^ Adjusted for age, sex, marital status, insurance status, educational level, poverty level, health status, internet use, and having usual source of care.

## Discussion

For patients with LEP, we found not only telehealth access disparities but also worse video visit experience. Additionally, characteristics of video and telephone visit users differed by English proficiency. Worse video visit experience may be associated with challenges in integrating interpreters into telehealth visits or perceived effectiveness by both clinicians and patients.^4^ Patients, especially those with LEP, prefer in-person care due to anxiety with self-evaluation without a medical professional.^5^ Digital barriers (eg, lack of affordable broadband/devices, unavailable translated portals, and limited digital literacy and support) may also play a role.^6^

Study limitations include reliance on self-reported telehealth use, focus on California, and inability to control for clinician factors that may affect care experience. Future work may evaluate the potential of digital navigators in improving the video visit experience. These findings highlight access to telephone visits alongside needed improvements to video visits for patients with LEP.

## Notes

Supplement 1.
eAppendix.

Supplement 2.
Data Sharing Statement

## References

[1] Rodriguez JA, Saadi A, Schwamm LH, Bates DW, Samal L. Disparities in telehealth use among California patients with limited English proficiency. Health Aff (Millwood). 2021;40(3):487-495. doi:10.1377/hlthaff.2020.00823 33646862

[2] Carrasquillo O, Orav EJ, Brennan TA, Burstin HR. Impact of language barriers on patient satisfaction in an emergency department. J Gen Intern Med. 1999;14(2):82-87. doi:10.1046/j.1525-1497.1999.00293.x 10051778

[3] UCLA Center for Health Policy Research. Accessed March 21, 2023. https://healthpolicy.ucla.edu/chis/data/Pages/GetCHISData.aspx

[4] Guetterman TC, Koptyra E, Ritchie O, . Equity in virtual care: a mixed methods study of perspectives from physicians. J Telemed Telecare. 2023;1357633X231194382. doi:10.1177/1357633X231194382 37641207

[5] Nguyen MT, Garcia F, Juarez J, . Satisfaction can co-exist with hesitation: qualitative analysis of acceptability of telemedicine among multi-lingual patients in a safety-net healthcare system during the COVID-19 pandemic. BMC Health Serv Res. 2022;22(1):195. doi:10.1186/s12913-022-07547-9 35164746 PMC8842908

[6] Arcury TA, Quandt SA, Sandberg JC, . Patient portal utilization among ethnically diverse low income older adults: observational study. JMIR Med Inform. 2017;5(4):e47. doi:10.2196/medinform.8026 29138129 PMC5705857

