# Supplementary material for: Telehealth Experience Among Patients With Limited English Proficiency
Source: JAMA Netw Open. 2024 May 9;7(5):e2410691. doi: 10.1001/jamanetworkopen.2024.10691 (PMC11082683; doi:10.1001/jamanetworkopen.2024.10691)
Supplement: Supplement 1. — eAppendix. [file jamanetwopen-e2410691-s001.pdf]

Supplemental Online Content

Rodriguez JA, Khoong EC, Lipsitz SR, Lyles CR, Bates DW, Samal L. Telehealth experience among patients with limited English proficiency. *JAMA Netw Open*. 2024;7(5):e2410691. doi:10.1001/jamanetworkopen.2024.10691

eAppendix.

This supplemental material has been provided by the authors to give readers additional information about their work.

© 2024 Rodriguez JA et al. *JAMA Network Open*

eAppendix.

A. Survey Questions
   a. Telehealth use
      i. During the past 12 months, did you receive care from a doctor or health professional through a video or telephone conversation rather than an office visit?
   b. Visit experience
      i. Video
         1. How would you rate the overall experience of your most recent video visit compared to an in-person visit? Would you say the video visit was...
      ii. Phone
         1. How would you rate the overall experience of your most recent phone visit compared to an in-person visit? Would you say the phone visit was...

© 2024 Rodriguez JA et al. *JAMA Network Open*
