# Supplementary material for: Telehealth Experience Among Patients With Limited English Proficiency
Source: JAMA Netw Open. 2024 May 9;7(5):e2410691. doi: 10.1001/jamanetworkopen.2024.10691 (PMC11082683; doi:10.1001/jamanetworkopen.2024.10691)
Supplement: Supplement 2. — Data Sharing Statement [file jamanetwopen-e2410691-s002.pdf]

Data Sharing Statement

Rodriguez. Telehealth Experience Among Patients With Limited English Proficiency. *JAMA Netw Open*. Published May 09, 2024. doi:10.1001/jamanetworkopen.2024.10691

Data
Data available: No

Additional Information
Explanation for why data not available: The data is available directly from the California Health Interview Survey
